# Building age-friendly communities through resource allocation and governance innovation under urban–rural integration: evidence from Sichuan, China

**DOI:** 10.3389/fpubh.2026.1858630

**Published:** 2026-07-20

**Authors:** Jia Ren, Xiao Wang

**Affiliations:** School of Digital Economics & Management, Mianyang Teachers’ College, Mianyang, Sichuan, China

**Keywords:** age-friendly communities, digital inclusion, governance innovation, resource allocation, urban–rural integration

## Abstract

Amid rapid population aging and persistent urban–rural disparities, age-friendly communities (AFCs) have become an important vehicle for strengthening community-level capacity to care for older adults and promoting urban–rural integration. Using Sichuan Province in western China as a case study, this paper examines three contrasting community types: an urban core community, an urban–rural fringe community, and a rural community. Drawing on policy document analysis, field observations, community operational materials, and 55 semi-structured interview notes from three anonymized case communities, this study investigates how resource allocation, governance arrangements, and smart technology shape AFC development under conditions of urban–rural integration. The findings suggest that AFC development in the three cases was shaped by the interacting influences of resource allocation, governance innovation, and technological embedding. More effective resource allocation was associated with greater accessibility and responsiveness of services for older adults, while collaborative and adaptive governance innovation appeared to support service continuity and organizational resilience. Smart technology was more likely to contribute to service efficiency when it was embedded in local governance networks and accompanied by measures supporting digital inclusion. The cross-case comparison points to three context-dependent development pathways across urban, urban–rural fringe, and rural communities. Overall, the evidence suggests that AFC development may be understood as a systemic and context-sensitive process requiring differentiated community strategies to improve the equity of services for older adults and support urban–rural integration in western China.

## 1 Introduction

Global demographic change is increasingly shaped by two interrelated challenges, rapid population aging and persistent urban–rural disparities. By 2050, one in six people worldwide is projected to be aged 65 years or older (1). China exemplifies this trend, with accelerated population aging and increasingly pronounced inequalities in the provision of care services for older adults between urban and rural areas. By the end of 2025, the population aged 60 years and above had reached 323 million, accounting for 23.0% of the national population; of these, 224 million were aged 65 years and above, representing 15.9% of the total population (2). Sichuan Province, a populous agricultural province in western China, faces the dual pressures of population aging and urban–rural integration. By the end of 2024, its population aged 60 years and above had reached 20.58 million, accounting for 24.6% of the provincial population, markedly above the national average for the same year (3). At the same time, Sichuan’s urban–rural dual structure remains pronounced: approximately 60% of high-quality medical and elder care resources are concentrated in major urban centers, whereas rural areas continue to face inadequate service provision, shortages of professional personnel, and weak infrastructure (4).

The World Health Organization’s Global Network for Age-friendly Cities and Communities emphasizes that Age-Friendly environments should be accessible, inclusive, and adaptable to technological change, providing an important conceptual framework for addressing urban–rural imbalances in elder care (5). In 2020, China’s National Health Commission launched the “National Demonstration Age-friendly Communities” initiative, further underscoring the pivotal role of grassroots communities in coordinating urban–rural elder care resources and promoting service equalization (6). Within this policy context, localities across Sichuan have gradually undertaken practical explorations in AFC development. For example, Caotang Road Community in Chengdu has used smart terminals to provide emergency response and care services for the oldest old, Fengxiang Community in Qingbaijiang has developed a mutual-aid model in which younger older adults support older seniors, Qingshen County has addressed the challenge of meal assistance for rural older adults through neighborhood kitchens, and areas such as Langzhong and Xuanhan have expanded access to rural elder care through digital platforms and mobile services, thereby helping to overcome the “last mile” problem in service delivery. In some localities, idle school buildings and other underused assets have also been repurposed into village-level elder care facilities, generating a range of locally distinctive practices.

Despite these positive developments, AFC development in western China continues to face three major bottlenecks. First, resource allocation remains mismatched, idle rural collective land and urban public space are underutilized, while elder care facilities remain insufficient in quantity and unevenly distributed. Second, the digital divide remains substantial, older adults in rural areas generally have lower educational attainment and more limited access to smart elder care services. Third, governance remains fragmented, policy barriers and weak interdepartmental coordination impede the development of an integrated governance framework characterized by urban–rural linkage and multi-actor collaboration. These constraints not only reduce the overall effectiveness of elder care services but also hinder deeper urban–rural integration and coordinated regional development.

Existing scholarship has largely examined these issues in isolation. Research on AFC development tends to focus separately on urban or rural settings, whereas studies of urban–rural coordination rarely engage directly with the institutional and service carriers of elder care. Similarly, research on smart elder care has not been sufficiently integrated into broader processes of urban–rural development. Evidence remains particularly limited for western rural China, where population aging is more severe and resources are relatively constrained. More importantly, a systematic analytical framework integrating resource allocation, governance mechanisms, and smart technology has yet to be established, limiting both theoretical explanation and practical guidance for AFC development within an urban–rural coordination framework.

Against this backdrop, a key question for coordinated urban–rural development in the new era is how AFC development can serve as an entry point for addressing the uneven and inadequate provision of elder care across urban and rural areas through the dual drivers of optimized resource allocation and governance innovation. Using Sichuan Province as an empirical setting, this study addresses three questions: (1) What resource and institutional barriers shape the development of AFCs in urban and rural Sichuan? (2) How can smart technologies facilitate the flow of elder care resources between urban and rural areas? and (3) How can community governance innovation promote the synergistic development of age-friendliness and urban–rural coordination?

By examining the practical challenges and local experiences of Sichuan Province, this study investigates the developmental pathways and operational mechanisms of AFCs from the perspective of urban–rural coordination. It further develops a theoretical framework for understanding how smart-enabled AFCs can promote coordinated urban–rural development. In doing so, the study seeks to provide both theoretical insight and policy implications for regions pursuing the equalization of elder care services and integrated urban–rural development.

## 2 Literature review

### 2.1 Age-friendly communities

The World Health Organization (WHO) framework provides a normative foundation for AFCs, emphasizing the optimization of physical environments to better meet the needs of older adults. Early discussions focused primarily on the extent to which built-environment features could accommodate the physiological characteristics of ageing populations, particularly with regard to barrier-free design, transportation, housing, and other forms of physical infrastructure (7–9). As the field has evolved, scholarly attention has increasingly shifted toward older adults’ mobility, quality of life, psychological needs, and social embeddedness. Central to this shift is the objective of supporting ageing in place, sustained participation, and active ageing among older adults (10, 11). More recently, AFCs have come to be understood as comprehensive community systems that integrate improvements in the physical environment, stronger service support, and healthier social relationship networks. Such communities emphasize the interaction between individuals and their environments, encourage older adults to engage in community activities, volunteer service, and decision-making processes, and thereby strengthen their sense of belonging and self-worth (12–14).

In terms of evaluation, existing research has gradually moved beyond static and standardized indicator systems toward approaches that place greater emphasis on older adults’ subjective experiences and group differences. Ivan’s study showed that even within the same urban space, different groups of older adults may exhibit substantial variation in their perceptions of community friendliness (15). Jiang further refined the indicator system for AFCs and demonstrated that the community environment, health status, and social participation jointly shape the level of active ageing (16). Overall, the evaluation of AFCs has shifted from a sole focus on objective provision to greater attention to actual effectiveness in use, combining both objective and subjective dimensions. In this sense, assessment criteria are increasingly grounded not simply in the presence of facilities, but in whether these facilities can be translated into accessible, perceptible, and sustainable forms of support.

However, the existing literature has paid relatively limited attention to the logic of resource allocation, the mechanisms of interest distribution, and the processes of governance. In particular, in the context of ongoing urban–rural integration in China, further discussion is needed on how age-friendliness can be continuously produced through sustained resource investment and governance coordination. More specifically, it remains necessary to examine how age-friendly resources and services can transcend urban–rural boundaries and achieve coordinated provision across spaces, administrative levels, and social actors.

### 2.2 Urban–rural integration and the coordination of age-friendly services

As urban–rural integration has become incorporated into age-friendliness research, the central concern has shifted from whether communities are age-friendly in themselves to whether age-friendly resources are distributed equitably. Marked disparities persist between urban and rural communities in terms of age-friendly infrastructure, while fragmented public service provision and uneven policy implementation capacity remain significant challenges (17). Beyond material inequalities, the institutional boundaries produced by the long-standing urban–rural dual structure constitute a deeper constraint on the coordination of age-friendly services. AFC planning spans multiple policy domains, including housing and urban–rural development, civil affairs, health, and transportation; however, the policy priorities and implementation logics across these sectors are not always aligned (18). Existing research indicates that effective age-friendly development depends on the integrated allocation of service resources, and that cross-sector collaboration, interest coordination, and multi-actor participation are essential to the effective operation of complex public service systems (19, 20).

Importantly, urban–rural coordination should not be understood as the simple equal distribution of resources. More fundamentally, the effectiveness of age-friendly development depends on whether resources can be transformed into actual accessibility, affordability, and usability (21). To advance the coordinated development of age-friendly services across urban and rural areas, governance innovation grounded in cross-sector collaboration and multi-stakeholder participation has been identified as an indispensable driving force. A substantial body of literature has shown that in areas involving spatial equity, such as public transportation planning, housing affordability, and subsidies for vulnerable groups, collaborative governance mechanisms can improve the efficiency of resource use and enhance service equity, thereby helping rural older adults gain equal access to basic public services and everyday support (22, 23).

Although the existing literature has acknowledged the significance of urban–rural disparities, it still lacks a systematic explanation of several key issues: how resources flow between urban and rural areas, how actors at different levels coordinate service allocation, how the logic of resource allocation shifts from quantitative expansion to precise matching, and how equity and efficiency can be balanced within institutional design.

### 2.3 Smart technology empowerment

In recent years, propelled by the rapid development of smart technologies, the advancement of urban–rural integration strategies, and a growing emphasis on equity, the integration of technology into community governance has emerged as a new driver of AFC development. This process has substantially improved the precision and sustainability of service provision (24, 25). On the one hand, given disparities among older adults in health status, family support, and digital literacy, the coordination of public services must shift toward equitable accessibility supported by differentiated interventions (26, 27). On the other hand, the application of technologies such as the Internet of Things, big data, and intelligent sensing can enhance service efficiency and optimize resource allocation within communities.

However, existing research suggests that although technological embedding can facilitate information connectivity, it does not automatically result in greater age-friendliness. Building urban–rural coordinated services for older adults through technological systems involves multiple stakeholders, including civil affairs, healthcare, housing, transportation, and community organizations. Without clear arrangements for data sharing, responsibility allocation, and governance authority, technical platforms may remain limited to simple information aggregation and fail to develop into effective collaborative mechanisms (28–30). Accordingly, whether smart technology can truly overcome spatial boundaries and empower AFCs in the context of urban–rural integration depends on its capacity to enable cross-community, cross-agency, and cross-regional service linkages through information sharing and network coordination. This process requires the simultaneous improvement of organizational workflows, governance structures, and interest-coordination mechanisms in order to achieve functional coupling between technological and governance systems.

In summary, although research on AFCs has expanded from physical environment optimization to broader dimensions such as social participation, public service, and active ageing, systematic analysis within the framework of urban–rural integration remains limited. From the dual perspective of resource allocation and governance innovation, this study focuses on the mechanisms of cross-sector resource allocation and the pathways of governance coordination in the development of AFCs. It further examines how technological empowerment can strengthen the equity, accessibility, and sustainability of services for older adults, thereby providing a more comprehensive theoretical framework for AFC development.

## 3 Research design

### 3.1 Analytical framework

This study conceptualizes the AFC as a comprehensive institutional vehicle that links resource allocation, governance innovation, smart technological empowerment, and service effectiveness. To examine this process, the study develops an analytical framework driven by the dual engines of resource allocation and governance innovation, with smart empowerment treated as a key embedded variable throughout.

At the driving level, the optimization of resource allocation and governance innovation constitutes the two core forces underlying AFC development. At the functional level, mechanisms such as spatial repurposing, the downward extension of services, organizational coordination, and platform connectivity facilitate the flow of resources between urban and rural areas, thereby improving the accessibility, precision of matching, and efficiency of utilization in services for older adults. At the transformational level, smart technology promotes the effective integration of resources and governance, further enhancing service accessibility, responsiveness, continuity, and equity. At the outcome level, these processes strengthen the capacity for AFC development and ultimately provide a grassroots foundation for urban–rural integration. Based on this analytical logic, the following propositions are proposed:

Proposition 1: Resource allocation optimization improves the coverage and accessibility of services for older adults in rural areas and urban-rural fringe communities by revitalizing idle spaces, extending public services downward, and reorganizing care resources for older adults.

Proposition 2: Governance innovation enhances the continuity, coordination, and precision of service provision for older adults through cross-departmental collaboration, multi-actor participation, and platform-based governance.

Proposition 3: Smart technology is more likely to translate resource advantages into accessible community services when it is embedded in governance networks, connected to offline service delivery, and adapted to the digital literacy and support needs of older adults.

Proposition 4: Resource allocation and governance innovation do not operate as parallel processes; rather, they influence urban-rural integration through a chain mechanism reinforced by technological platform embedding and service transformation.

### 3.2 Case selection and sample profile

Case selection followed the principles of analytical relevance, variation, comparability, and data accessibility. The three cases were selected to represent contrasting positions along the urban–rural continuum and to capture variation in resource endowment, demographic structure, service accessibility, digital infrastructure, and governance capacity. Their classification was based on a combined contextual assessment of administrative location, spatial position, built-environment characteristics, population size and aging structure, availability of care facilities for older adults and professional services, digital service foundations, and local governance settings. Per capita income was not used as an independent classification criterion, although differences in economic and service-resource conditions were indirectly reflected in the availability of facilities, professional services, and digital support.

The urban core case was selected to examine AFC development in a high-density community with relatively mature service facilities and stronger smart-service capacity. The urban–rural fringe case was selected to capture a transitional governance context in which formal public services coexist with neighborhood mutual aid and participation by social organizations. The rural case was selected to examine how AFC development is promoted under conditions of dispersed settlement, limited professional resources, and greater reliance on village-level organizations and the downward extension of external resources.

To balance analytical transparency with confidentiality, the three case communities were anonymized as U1, F1, and R1. Because the combination of location, population structure, service facilities, and governance practices could make individual communities identifiable, contextual information is reported using approximate ranges and categorical descriptions rather than exact community names or precise administrative addresses. This approach maintains sufficient contextual visibility for cross-case analysis while reducing the risk of community identifiability (Table 1).

**Table 1:** Contextual profile of the anonymized case communities.

**Dimension**	**U1**	**F1**	**R1**
Case type	Urban core community	Urban–rural fringe community	Rural community
Spatial position	Located in a central urban built-up area with high residential density and relatively concentrated public service resources	Located in a transitional area between urban and rural systems, with mixed residential patterns and uneven access to urban services	Located in a township or village-level rural setting with relatively dispersed settlement patterns
Approximate geographical location	Central urban district of Sichuan Province	Peri-urban or urban–rural junction area of Sichuan Province	Township or village-level rural area of Sichuan Province
Administrative setting	Subdistrict-community governance structure with relatively mature grid-based management	Transitional governance structure involving subdistrict or township administration, community or village organizations, and social actors	Township-village governance structure relying mainly on village-level organizations and support from township-level authorities
Approximate resident population	Approximately 8,000–12,000 residents	Approximately 5,000–8,000 residents	Approximately 2,000–4,000 residents
Approximate share of residents aged 60 years and above	About 20–25% of the resident population	About 25–30% of the resident population	More than 30% of the resident population
Aging characteristics	Relatively high demand from the oldest-old, empty-nest older adults, older adults living alone, and older residents with chronic conditions	Mixed older population, including original residents, relocated residents, and older adults with diverse service needs	Deep aging tendency, with a relatively high proportion of left-behind, disabled, low-income, or empty-nest older residents
Priority care needs of older adults groups	Moderate to high number of older adults living alone, empty-nest older adults, and older adults with chronic diseases	Moderate number of older adults with diverse care needs, including relocated older residents and older adults requiring regular community support	High proportion of left-behind, empty-nest, disabled, semi-disabled, or low-income older adults requiring basic and continuous support
Main service facilities	Community service center, service for older adults station, health service point, meal-support site, activity room for older adults, smart service terminal, and platform-supported service arrangements	Community or village service station, mutual-aid space, activity room for older adults, volunteer service network, partial meal-support services, and limited platform-supported service arrangements	Village activity room, repurposed public space, basic service point for older adults, mobile medical or care resources for older adults, simplified smart service support where available
Service system profile	Relatively complete community-based service system for older adults, including assisted dining, health follow-up, emergency calling, home visits, cultural activities, and smart platform-supported demand registration or service dispatch	Mixed service system combining formal community services, social organization projects, volunteer activities, mutual-aid networks, and neighborhood-based support	Basic and low-cost service system relying on village-level coordination, mobile services, home visits, meal assistance, neighborhood mutual aid, and downward extension of township or county-level resources
Digital foundation	Relatively strong; smart terminals, digital registries, or platform-based service management are more accessible	Moderate but uneven; some digital tools are available, but actual use depends on staff, volunteers, and resident participation	Relatively weak; digital service use depends more heavily on village cadres, grid managers, family members, or mobile service teams
Key governance actors	Community committee, grid workers, health service institutions, care service providers for older adults, social organizations, volunteers, and family members	Community or village committee, social organizations, volunteer groups, mutual-aid networks, service providers, and residents’ representatives	Village committee, township government, grid managers, family caregivers, neighborhood mutual-aid actors, mobile medical or care service teams for older adults
Primary challenges	Fragmented service chains, high pressure on demand responsiveness, and the need to integrate smart platforms with offline services	Insufficient formal service supply, unstable mutual-aid mechanisms, and weak institutional interfaces between urban and rural resources	Shortage of professional service resources, limited facilities, low digital literacy, and weak sustainability of service provision
Analytical value	Observing how digital embedding and grid-based governance support refined services for older adults in high-density urban communities	Observing how formal governance, social organization participation, and neighborhood mutual aid are coupled in transitional communities	Observing how idle resource activation, village-level coordination, and downward resource extension support AFC development in resource-constrained rural settings

Case names were anonymized to reduce the risk of community identifiability. U1 refers to the urban core community, F1 to the urban–rural fringe community, and R1 to the rural community. The approximate population and aging ranges were derived from community operational materials and fieldwork verification, and are reported as ranges to protect community anonymity.

### 3.3 Data collection and analysis procedures

To address the research questions and enhance the methodological transparency of the case-based analysis, this study adopted a qualitative-dominant multiple-case study supported by triangulated data sources. Specifically, the study combined policy document analysis, community operational material analysis, field observations, semi-structured interviews, and cross-case comparison, thereby strengthening its explanatory capacity for complex governance processes.

The research was conducted in two stages. In the first stage, preliminary case mapping and documentary material collection were carried out in February 2025. This stage was mainly used to identify representative local practices of age-friendly community development in Sichuan Province and to clarify the policy and institutional context of services for older adults under the background of urban–rural integration. In the second stage, follow-up fieldwork, semi-structured interviews, community observations, and data verification were conducted between June 2025 and April 2026.

#### 3.3.1 Fieldwork setting and data sources

Three case communities in Sichuan Province, China, were selected to represent different spatial positions and governance conditions along the urban–rural continuum. These three cases were selected according to the principles of analytical relevance, variation, comparability, and data accessibility.

Four types of empirical materials were collected in this study. First, policy documents were used to reconstruct the institutional context of age-friendly community development, basic care services for older adults, smart care for older adults, grassroots governance, public service equalization, and urban–rural integration. These documents included national plans and guidelines, provincial policy documents issued by Sichuan Province, municipal-level implementation documents, and available community-level materials. Second, community operational materials were collected, including service manuals, project reports, platform descriptions, service flowcharts, work summaries, service records, and publicly available promotional materials. These materials were used to identify how policies related to age-friendly communities were translated into community-level service arrangements and governance practices. Third, field observations were conducted in community service stations, meal-support sites, health service points, activity spaces for older adults, smart service terminals, village activity rooms, and age-friendly public spaces. Observation notes focused on spatial arrangements, service processes, actor interactions, platform use, and older residents’ actual use of community services. Fourth, semi-structured interviews were conducted with key stakeholders and older residents to capture perceptions and experiences that were not fully reflected in formal documents.

Fieldwork data were not used as independent quantitative measurements. Instead, they served three analytical purposes. In this sense, fieldwork data were mainly used to answer how AFC-related services were organized and implemented in practice, rather than to produce statistical estimates. First, field observations were used to verify whether the practices described in policy documents and community operational materials were actually implemented in daily community settings. Second, they helped capture service processes that were not fully documented in written materials, such as how older residents accessed meal assistance, health management, emergency response, home visits, and smart care services for older adults. Third, field observations were used to triangulate interview materials and documentary evidence, thereby strengthening the empirical basis for cross-case comparison.

In each case community, two to four field visits were conducted during the fieldwork period, depending on the accessibility of the site and the availability of community staff, service providers, and older residents. The field visits included non-participant observations of community service stations, meal-support sites, health service points, activity spaces for older adults, smart service terminals, village activity rooms, and age-friendly public spaces. During these visits, the research team recorded observation notes on the spatial layout of service facilities, the actual use of age-friendly spaces by older residents, the interaction between community workers and service users, the operation of smart service devices or platforms, and the connection between formal services and informal mutual-aid practices. These observation notes were used not as quantitative indicators, but as contextual and triangulating materials to support the interpretation of interview data and documentary evidence.

#### 3.3.2 Semi-structured interviews and participant recruitment

Semi-structured interviews were conducted with participants who were directly involved in or affected by age-friendly community development. Participants were recruited through purposive sampling, supplemented by snowball sampling where necessary. The sampling strategy aimed to include actors with different roles in community-based care for older adults and community governance, including community officials, neighborhood or village committee staff, care service providers for older adults, medical or health service personnel, representatives of social organizations or volunteer groups, and older residents.

Older residents were eligible for inclusion if they were aged 60 years or above, had lived in the community for at least 6 months, and had experience using or being exposed to community-based care for older adults. To capture diverse experiences, older participants were selected to reflect variation in age, gender, living arrangement, health status, mobility status, service needs, and digital literacy.

A total of 55 semi-structured interview notes were included in the analysis. The interviewees included 11 community officials or neighborhood/village committee staff, 8 care service providers for older adults, 10 medical or health service personnel, 6 representatives of social organizations or volunteer groups, and 20 older residents. The distribution of interviewees across the three case communities is shown in Table 2.

**Table 2:** Distribution of interviewees by case community and participant group.

**Case code**	**Case type**	**Community officials/ neighborhood or village committee staff**	**Care service providers for older adults**	**Medical/ health service personnel**	**Leaders or representatives of social organizations/ volunteer groups**	**Older residents**	**Total**
U1	Urban core community	6	5	6	3	10	30
F1	Urban–rural fringe community	3	2	3	2	6	16
R1	Rural community	2	1	1	1	4	9
Total	**—**	**11**	**8**	**10**	**6**	**20**	**55**

The unequal distribution of interviews across the three cases reflected differences in site accessibility, stakeholder availability, and the density of relevant service actors in each community. Interview counts were not used as analytical weights in the cross-case comparison. Instead, the cases were compared on the basis of recurring themes, contextual conditions, and triangulated evidence from interviews, field observations, community operational materials, and policy documents.

The interview guide was developed according to the analytical framework of this study and covered four primary dimensions: resource allocation, governance innovation, smart empowerment, and service effectiveness. Interviews with community officials, service providers, medical personnel, and social organization representatives focused on resource mobilization, service organization, interdepartmental coordination, platform use, implementation barriers, service referral, and feedback mechanisms. Interviews with older residents focused on service accessibility, perceived usefulness of community services, difficulties in using smart technologies, daily care needs, social participation, and satisfaction with age-friendly community development.

Each interview lasted approximately 30–60 min. With participants’ informed consent, interviews were audio-recorded where permitted, resulting in 16 audio recordings. The 16 audio-recorded interviews were transcribed verbatim. For interviews that were not audio-recorded, detailed contemporaneous notes were expanded immediately after the interview and used as interview-note excerpts rather than verbatim transcripts. All interview materials were organized in Chinese, anonymized, and assigned codes according to participant type and case community. Selected quotations used in the manuscript were translated into English by the research team and checked against the original Chinese notes or transcripts to ensure semantic accuracy.

Thematic sufficiency was considered during the later stage of fieldwork and preliminary coding. After approximately 45 interviews had been completed, repeated interviews and observations across the three case communities indicated that no substantially new first-order categories were emerging regarding resource allocation barriers, service fragmentation, governance coordination, digital exclusion, service accessibility, or community-based support mechanisms. The remaining 10 interviews were mainly used to confirm and refine the patterns that had already been identified. Therefore, the empirical materials were considered sufficient for the purposes of thematic analysis and qualitative cross-case comparison.

#### 3.3.3 Coding, thematic analysis, and cross-case comparison

Data analysis followed a coding strategy that combined deductive and inductive approaches. First, the research team developed an initial codebook deductively based on the research questions and analytical framework, identifying four primary coding dimensions: resource allocation, governance innovation, smart empowerment, and service effectiveness. Second, open coding was conducted to identify empirical categories from interview notes, field notes, policy documents, and community operational materials. These categories included spatial resource activation, facility mismatch, service-chain fragmentation, downward resource extension, mutual-aid networks, platform-based demand identification, digital exclusion, proxy assistance, interdepartmental coordination, responsibility ambiguity, and closed-loop service feedback. Third, axial coding was used to examine relationships among categories and to generate higher-level themes. Finally, cross-case matrices were constructed to compare U1, F1, and R1, with the aim of identifying both common mechanisms and differentiated pathways in age-friendly community development.

The coding process was conducted in three steps. In the first step, the research team repeatedly read all empirical materials to identify descriptions related to care resources for older adults, service delivery, digital technology use, governance coordination, and older residents’ service experiences. In the second step, codes were grouped into broader categories under the four analytical dimensions. In the third step, the three case communities were compared horizontally to examine how similar mechanisms operated under different resource endowments, demographic structures, governance foundations, and digital conditions.

Thematic analysis was used to connect empirical evidence with the analytical propositions of this study. Evidence related to resource allocation was used to examine how spatial resources, service facilities, and professional service capacity were reorganized at the community level. Evidence related to governance innovation was used to analyze how government departments, community organizations, medical institutions, social organizations, volunteers, families, and older residents participated in age-friendly community development. Evidence related to smart empowerment was used to examine whether and how digital platforms, smart terminals, and service dispatch mechanisms improved service accessibility and responsiveness. Evidence related to service effectiveness was used to assess whether community-based services for older adults became more accessible, responsive, continuous, and equitable.

Representative interview excerpts were selected to illustrate the major themes in the Results section. These excerpts were not treated as isolated anecdotal evidence, but were interpreted together with policy documents, field observations, and community operational materials. This approach helped establish an evidence trail between the empirical materials and the study’s claims regarding resource mismatch, service fragmentation, digital exclusion, governance coordination, and differentiated pathways of age-friendly community development (Table 3).

**Table 3:** Coding framework for empirical analysis.

**Primary dimension**	**Sub-themes**	**Main data sources**	**Analytical purpose**
Resource allocation	Spatial resource mismatch; idle space activation; facility distribution; downward service extension; service supply–demand matching	Policy documents; community materials; field observations; interviews	To examine how resources were transformed into community-based care for older adults capacity
Governance innovation	Multi-actor participation; departmental coordination; community grid management; social organization involvement; mutual aid; responsibility interface; feedback mechanisms	Interviews; operational materials; field observations	To analyze how governance arrangements shaped service continuity and coordination
Smart empowerment	Digital registry; smart terminals; platform dispatch; one-touch calling; health monitoring; digital literacy; proxy assistance; digital exclusion	Field observations; platform materials; interviews	To assess whether smart technology improved accessibility, responsiveness, and inclusion
Service effectiveness	Service accessibility; responsiveness; continuity; equity; perceived usefulness; older residents’ satisfaction	Interviews; field observations; cross-case comparison	To evaluate the practical effects and differentiated pathways of AFC development

#### 3.3.4 Trustworthiness and ethical considerations

Several measures were adopted to strengthen the rigor and trustworthiness of the qualitative analysis. First, data triangulation was conducted across policy documents, community operational materials, field observations, and semi-structured interview notes, thereby reducing the risk of relying solely on formal documents or single-source accounts. Second, two researchers independently coded a subset of the materials and discussed coding discrepancies until agreement was reached. The coding framework was then revised and applied to the full dataset. Third, an audit trail was maintained to document the development of codes, categories, themes, and cross-case comparison matrices. Fourth, the research team repeatedly checked preliminary interpretations against field notes and community materials to avoid over-interpretation and to ensure that the findings were grounded in empirical evidence. These procedures helped improve the credibility, dependability, confirmability, and transferability of the qualitative analysis.

This study followed ethical principles for research involving human participants. Before each interview, the research team informed participants of the research purpose, the voluntary nature of participation, the expected use of the materials, confidentiality measures, and their right to withdraw at any time. Verbal informed consent was obtained from all participants. During transcription, note organization, coding, and analysis, personal identifiers were removed, and both participants and case communities were anonymized. The authors confirmed the ethical requirements for this low-risk qualitative study with the university research ethics committee. As the study involved only minimal-risk interviews and field observations, did not collect sensitive personal medical information, and used anonymized data for academic analysis, it was conducted in accordance with the institution’s ethical guidance for minimal-risk social science research. No formal ethics approval number was assigned by the committee.

## 4 Empirical analysis

In presenting the empirical findings, this section combines cross-case thematic analysis with within-case evidence. Interview excerpts are used to illustrate how different actors perceived resource allocation, service accessibility, smart technology use, and governance coordination in the three anonymized case communities. The excerpts are identified by case code and participant type. CS denotes community staff, SP service provider, MP medical personnel, VG volunteer or social organization representative, and OR older resident. Interview quotations were translated from Chinese into English and checked against the original interview notes or transcripts to preserve the original meaning (Table 4).

**Table 4:** Evidence matrix connecting analytical themes, within-case operational evidence, and representative interview excerpts.

**Analytical theme**	**Case evidence**	**Within-case operational evidence**	**Representative interview excerpt**	**Analytical interpretation**
Spatial and facility resource mismatch	U1 and R1	In U1, community service rooms, activity spaces for older adults, health service points, and care stations for older adults were available, but older residents increasingly required nearby, home-based, and individualized services. In R1, village activity rooms and other public spaces existed, but their service functions were limited by irregular opening hours, insufficient staffing, and weak professional support.	“We have service rooms and activity spaces, but many older residents now need faster help at home, not only activities in the center” (U1-CS-02). “There is a village activity room, but it is not open every day, and there are not many services for people like us” (R1-OR-03).	Resource mismatch was not only reflected in the unequal distribution of facilities, but also in the weak conversion of existing spaces into accessible, continuous, and demand-responsive capacity to care for older adults.
Service-chain fragmentation	U1 and F1	In U1, meal assistance, health checks, home visits, and emergency response were available but were often organized by different actors. In F1, professional service projects and volunteer-based activities were not always connected through a stable service process.	“For meals, health checks, and home visits, we often ask different people. Sometimes we do not know who should help first” (U1-OR-05). “We provide some services, but they are not always arranged together” (F1-SP-01).	Service items could be formally available without forming a clear and continuous support pathway. In F1, fragmentation was reflected in the weak connection between professional projects and community-based support.
Digital exclusion in smart care for older adults	U1 and R1	In U1, smart terminals and digital records were more visible, but some older adults still needed assistance from community workers or family members. In R1, weaker digital literacy and dispersed settlement patterns made older residents more dependent on village cadres, grid managers, and family members for access to services.	“I can use some simple functions, but when the steps are complicated, I still ask community staff to help me” (U1-OR-06). “I do not know how to use the phone for these services. If I need something, I still call the village cadre” (R1-OR-02).	Digital exclusion was not limited to the absence of devices. It was also shaped by older adults’ operational ability, confidence, usability barriers, and the availability of interpersonal support.
Governance fragmentation	U1, F1, and R1	In U1, coordination became time-consuming when older residents required several services. In F1, social organization and volunteer participation was more active during project periods but became less stable afterward. In R1, youth out-migration weakened family support and increased pressure on village-level actors.	“Different departments have their own tasks. When an older person needs several services, coordination takes time” (U1-CS-03). “There are more activities when a project is running, but after the project ends, it becomes harder to keep everyone involved” (F1-VG-01).	Coordination costs, project dependence, and unclear responsibility interfaces limited service continuity. The cases suggest that temporary mobilization did not necessarily develop into routinized institutional cooperation.
Multi-actor coordination	U1 and F1	In U1, community staff linked older residents with hospitals, service providers, volunteers, and families. In F1, service providers relied on community workers and neighborhood networks to identify real service needs.	“The community cannot do everything alone. We have to connect hospitals, service providers, volunteers, and families” (U1-CS-01). “Professional organizations can provide some services, but we still depend on community workers to tell us who really needs help” (F1-SP-02).	Community organizations played a bridging role between formal resources and everyday needs. However, this role also concentrated coordination responsibilities at the community level and depended on sufficient staffing, communication, and institutional support.
Mutual-aid governance and local relational resources	F1 and R1	In F1, younger older adults and volunteers supported visits, reminders, activity participation, and the identification of emerging needs. In R1, neighbors, village cadres, and grid managers were often the first point of contact for routine difficulties.	“Some older residents do not need professional care every day, but they need someone nearby to check on them” (F1-VG-02). “When I have a problem, I usually look for the village cadre or a neighbor. They are close and know our situation” (R1-OR-03).	Mutual aid extended the reach of formal services for frequent, low-professional-intensity needs. Its continuity was nevertheless uneven and actor-dependent, and it could complement but not replace nursing, rehabilitation, medical referral, or other specialized services.
Smart technology as a service coordination tool	U1	In U1, digital platforms and smart terminals were used for demand registration, risk identification, classified management, task dispatch, health follow-up, emergency calling, and feedback tracking. The typical process included information registration, need classification, service matching, offline delivery, and follow-up updating.	“Digital records help us know which older residents live alone or need regular attention, but the information must be updated; otherwise, it is not useful for daily work” (U1-CS-04). “When health risk records are available on the platform, we can contact the community more quickly and arrange follow-up for older residents who need attention” (U1-MP-01).	Smart technology appeared more likely to support service responsiveness when platform data were continuously updated and embedded in routine governance workflows. Its contribution lay in connecting demand identification with offline service action.
Digital inclusion through human mediation	F1 and R1	In F1, volunteers helped older residents scan codes, make appointments, and gradually become familiar with digital services. In R1, village cadres and grid managers often helped older residents complete online registration, enter information, and contact service providers.	“We often help older residents scan codes or make appointments. After doing it several times, some of them feel less afraid” (F1-VG-02). “Many older residents cannot complete online registration by themselves, so we help them enter information and contact service providers” (R1-CS-01).	Digital inclusion was a relational and organizational process rather than a purely technical outcome. Smart services appeared more usable for older residents when supported by volunteers, community workers, grid managers, family members, and village cadres.

CS denotes community staff, SP service provider, MP medical personnel, VG volunteer or social organization representative, and OR older resident. U1 refers to the urban core community, F1 to the urban–rural fringe community, and R1 to the rural community. Representative excerpts were translated from Chinese into English and checked against the original interview notes or transcripts to preserve the original meaning.

### 4.1 Constraints on AFC development under urban–rural integration

As a major populous and agricultural province in western China, Sichuan is characterized by pronounced population ageing and substantial spatial disparities between urban and rural areas. In this context, the development of AFCs is not merely a matter of upgrading community services; rather, it reflects the combined effects of the long-standing urban–rural dual structure, the uneven distribution of grassroots public services, fragmented governance arrangements, and the limited adaptability of technology to older populations. Evidence from fieldwork and case summaries indicates that current AFC development is constrained by four interrelated dimensions: resource misallocation, service fragmentation, digital exclusion, and fragmented governance.

#### 4.1.1 Spatial and facility resource imbalances

The provision services for older adults across Sichuan exhibits a clear pattern of centralized concentration. High-quality medical, rehabilitation, and professional care resources are largely concentrated in urban cores and economically developed counties, whereas rural areas and urban–rural fringe communities continue to face inadequate service stations, weak infrastructure, and lagging age-friendly retrofitting. At the same time, many communities do not entirely lack usable space; rather, existing assets, such as public spaces, vacant school buildings, and village activity centers, have not yet been effectively converted into service carriers. This has created a structural contradiction in which resources exist, yet service provision remains insufficient. The primary constraint on AFC development is therefore not an absolute scarcity of resources, but a mismatch between resource volume, spatial distribution, and actual service demand.

Field interview notes further confirmed that spatial and facility constraints differed across the three cases. In U1, interview notes from a community staff member indicated that although service rooms and activity spaces were available, many older residents increasingly needed faster home-based support rather than only center-based activities (U1-CS-02). This suggests that resource mismatch in the urban case was reflected in the gap between existing facilities and more individualized service needs. In R1, interview notes from an older resident indicated that the village activity room was not open every day and provided only limited services for older residents (R1-OR-03). This shows that the rural problem was not only the lack of physical space, but the weak conversion of existing space into regular and usable services for older adults.

#### 4.1.2 Fragmented services for older adults delivery

AFC development involves more than physical adaptation; it also requires the integrated embedding of multiple service functions, including assisted dining, nursing care, emotional support, health monitoring, and emergency assistance. The field interview notes showed that older residents often experienced services as separate rather than integrated. In U1, interview notes from an older resident indicated that meal assistance, health checks, and home visits were usually handled by different actors, and residents sometimes did not know whom to contact first (U1-OR-05). This illustrates how service fragmentation reduced the convenience and continuity of services for older adults, even when different service items were formally available. The field interview notes from a service provider in F1 suggested that formal service projects and volunteer-based mutual-aid activities were not always planned or arranged together (F1-SP-01). This indicates that the key problem in the urban–rural fringe case was not only insufficient service supply, but also the lack of stable coordination among different service channels.

#### 4.1.3 Digital exclusion in smart care for older adults

In recent years, many areas in Sichuan have actively promoted smart community and care platforms for older adults, seeking to integrate functions such as one-touch calling, health monitoring, service dispatch, online consultation, and emergency response into community governance. However, practical experience shows that technological embedding does not automatically generate greater age-friendliness. In some cases, a structural contradiction has emerged in which platforms are technologically advanced, yet actual utilization remains limited.

Case findings indicate that the digital divide constitutes a widespread barrier. The first dimension concerns access to equipment: urban communities are more likely to obtain smart terminals and platform support, while rural communities often remain constrained by weak network infrastructure and limited terminal maintenance. The second dimension concerns user capability: even where platforms are available, older adults differ markedly in their ability to understand and operate digital devices according to age, educational attainment, and physical condition. The third dimension concerns relational support: for many older adults, the usability of technology depends less on the device itself than on whether community workers, volunteers, adult children, or neighbors can provide assistance and accompaniment.

The field interview notes further showed that digital exclusion was not limited to the absence of devices. In U1, smart terminals and digital records were more visible, but interview notes from an older resident indicated that some older adults still needed help from community workers or family members when using smart services (U1-OR-07). This finding suggests that smart technology may remain inaccessible when older adults are unable to operate it independently. This problem was more pronounced in R1. Field interview notes from a rural older resident suggested that some older adults did not know how to use mobile phones to access community-based services and still preferred to contact village cadres when they needed assistance (R1-OR-02). This indicates that, in rural settings, digital exclusion was closely related to limited digital literacy and continued reliance on familiar offline contacts. Field interview notes from a grid manager in R1 further indicated that many older residents could not complete online registration by themselves, so village cadres or grid workers usually helped them enter information and contact service providers (R1-CS-01). This account suggests that digital inclusion depended on human mediation and proxy assistance, rather than technology coverage alone.

#### 4.1.4 Gaps in multi-actor governance

Compared with visible constraints in resources and technology, fragmented governance represents a deeper institutional barrier to the sustainable operation of AFCs. AFC development involves multiple stakeholders, including departments responsible for civil affairs, health, housing, social security, and agriculture, as well as different levels of local government. Although policy objectives broadly converge around active ageing, these actors often differ in resource allocation priorities, evaluation criteria, and administrative boundaries. In the context of urban–rural integration, the downward flow and shared use of resources require stable and sustained coordination across administrative levels and sectoral boundaries. Without such coordination, resource optimization may remain confined to short-term project implementation, while technological empowerment may be reduced to information collection, failing to evolve into a stable institutional arrangement.

Fieldwork indicated that governance fragmented was also reflected in unclear responsibility boundaries among families, communities, service providers, and government departments. In U1, interview notes from a community staff member suggested that different departments had their own tasks, and coordination became time-consuming when an older resident needed several types of services at the same time (U1-CS-03). This shows that fragmented governance can delay service responses even in communities with relatively abundant resources. In F1, field interview notes from a volunteer representative indicated that service activities were more frequent during project implementation, but became harder to sustain after the project ended (F1-VG-01). This suggests that without stable institutional support, mutual-aid practices and social organization participation may remain temporary rather than normalized. In R1, interview notes from a village worker suggested that many young adults worked outside the village, making it difficult for older residents to rely fully on family care (R1-CS-02). This finding shows that the weakening of family-based support has increased pressure on community- and village-level public services.

### 4.2 Resource reorganization in AFC development

#### 4.2.1 Activating existing spatial resources

Evidence from the three case communities indicates that the revitalization of existing resources not only lowers the threshold for service provision, but also enhances the feasibility of embedding services within community settings. Compared with constructing large, standalone care institutions for older adults, converting existing community spaces into small-scale, embedded service sites is more closely aligned with older adults’ daily activity radius. These sites are easier for older residents to identify and use, while also helping to preserve internal community social networks, thereby improving the local accessibility of basic services.

The field interview notes repeatedly emphasized the importance of embedded service spaces. In U1, interview notes from an older resident indicated that nearby service points were more likely to be used, while distant facilities were less attractive to older residents with limited mobility (U1-OR-04). This finding suggests that spatial accessibility is essential for transforming facilities into services that older residents actually use. In R1, field interview notes from a village worker suggested that reusing existing public rooms was a more realistic option than building a new care center for older adults in the short term (R1-CS-01). This finding supports the argument that rural AFC development often depends on low-cost spatial activation rather than large-scale new construction.

#### 4.2.2 Reorganizing service supply

Comparative analysis further shows that the reorganization of service units and the establishment of embedded service networks constitute key organizational forms of resource allocation optimization. Urban communities can rely on community service centers, health stations, and grid-based management systems to establish multiple service nodes around a central platform and thereby deliver home-based care. By contrast, urban–rural fringe and rural communities depend more heavily on village-level organizations, neighborhood spaces, and mobile service teams to form an operational model that combines fixed service points with roving support. Although these pathways differ, they share a common feature: service provision is no longer institution-centered, but instead organized around older adults’ daily life circles, placing core support within a short-response range. In this way, service resources that were previously dispersed across spaces and administrative sectors are integrated into a continuous community-based care support network for older adults.

From the perspective of urban–rural integration, the significance of reorganizing service networks lies in its provision of an organizational foundation for the cross-spatial flow of resources. High-quality urban resources can be extended to grassroots communities through platforms, referral systems, mobile clinics, and professional support. At the same time, mutual-aid relations and localized organizational capacity in rural communities can be incorporated into a broader service system through networked coordination. Resource allocation optimization is therefore not a simple matter of spatial redistribution, but a dynamic process of resource circulation and functional enhancement achieved through network reconstruction.

#### 4.2.3 Precision matching of care resources for older adults

The study further finds that, given the uneven distribution of high-quality resources in Sichuan’s AFC development, the cross-sector flow of resources and the downward extension of services represent critical avenues for optimization. Case evidence shows that some localities have introduced professional medical care, rehabilitation guidance, social work services, and training resources into rural communities through county-level coordination, medical alliances, mobile clinic mechanisms, paired assistance, and the downward extension of social organization projects. Through institutional design and platform connectivity, the spatial fixity of resources within administrative and geographic boundaries can be reduced, enabling professional service capacity originally concentrated in urban centers to diffuse to grassroots communities. This reflects a broader shift in the logic of resource allocation under urban–rural integration, from institutional concentration to community embedding, and from static distribution to dynamic mobility.

At the practical level, allocation priorities vary across community types. Urban communities place greater emphasis on emergency response, refined care, and technological platform connectivity to meet the dynamic needs of the oldest-old, older adults living alone, and those with chronic illnesses. Urban–rural fringe communities focus more on the downward extension of resources and connective supply, strengthening the referral and organizational capacity of grassroots communities while absorbing urban resources. Rural communities prioritize low-cost, localized, and mutual-aid-based services in order to address high-frequency basic needs such as meal assistance, home visits, daily care, and emotional companionship. Demand-oriented precision matching therefore appears to be an important condition influencing whether resource reorganization can be translated into more accessible and responsive community services.

The field interview notes showed that service needs differed across the three community types. In U1, interview notes from a medical worker indicated that some older residents living alone and those with chronic diseases required closer follow-up and faster responses from community-based health and care services for older adults (U1-MP-02). This suggests that urban AFC development requires more refined demand identification and health-related follow-up. In F1, field interview notes from a volunteer representative emphasized that some older residents did not require professional care every day, but needed nearby neighbors or volunteers to check on them regularly (F1-VG-02). This finding indicates that mutual aid can supplement formal services when older residents’ needs are frequent but not highly professional. In R1, interview notes from an older resident suggested that meal assistance, home visits, and having someone to contact when difficulties arose were among the most useful forms of support (R1-OR-04). This finding shows that rural resource matching should prioritize basic, high-frequency, and easily accessible services.

### 4.3 Governance innovation in AFC development

#### 4.3.1 Broadening governance participation

Under traditional models, community-based care for older adults has often been treated as an extension of welfare provision led primarily by government departments. In practice, however, no single actor can effectively manage the multiple tasks involved in AFC development, including spatial renovation, service organization, health care, emergency support, and social mobilization. Evidence from the three cases suggests that community-based care for older adults relied on the participation of government departments, community organizations, medical institutions, service providers, social organizations, volunteers, families, and older residents, although the stability and effectiveness of their cooperation varied across cases.

The field interview notes indicated that community-based care for older adults depended on cooperation among multiple actors, although such cooperation was not inherently stable or equally effective across the three cases. In U1, a community staff member explained that the community could not provide all services for older adults alone and therefore had to coordinate with hospitals, service providers, volunteers, and family members (U1-CS-01). This account illustrates both the community’s bridging role and the additional coordination workload created by the involvement of multiple actors. In F1, a service provider noted that professional organizations could provide services for older adults but still depended on community workers to identify which older residents actually required assistance (F1-SP-02). This dependence facilitated more accurate demand identification, but it also meant that coordination could weaken when community workers were overburdened or when external projects ended. As one volunteer representative observed, “There are more activities when a project is running, but after the project ends, it becomes harder to keep everyone involved” (F1-VG-01). These findings suggest that multi-actor participation expanded service capacity, but the continuity of collaborative service provision remained conditional on stable institutional linkages, sustained resources, and clearly assigned coordination responsibilities.

The expansion of governance participation may therefore be understood as a gradual shift in the role of government and community organizations, from direct and relatively isolated service provision toward the organization and facilitation of collaborative relationships. However, incorporating more actors did not automatically result in stable collaboration. Its practical contribution depended on whether participating organizations could establish routinized communication, referral, resource-sharing, and responsibility-allocation arrangements.

#### 4.3.2 Integrating governance relationships

The findings suggest that governance relationships were being reorganized in three main respects, although the extent and stability of this process varied across the three cases. First, some forms of departmental coordination had developed through cross-sector linkages involving infrastructure, health care, care for older adults, digital platforms, and community governance. However, coordination remained time-consuming when an older resident required several types of support simultaneously. Second, communities increasingly acted as intermediaries connecting top-down government resources with bottom-up resident needs and coordinating horizontally with medical institutions, service providers, social organizations, and volunteers. This intermediary role facilitated service linkage but also concentrated substantial coordination responsibilities at the community level. Third, responsibility interfaces were becoming clearer in some service processes, while project-based provision, overlapping responsibilities, and differences in organizational priorities continued to create gaps in service continuity.

Governance innovation should therefore not be understood as a completed transition from fragmented governance to seamless collaboration. Rather, the three cases point to an uneven and ongoing process in which emerging collaborative relationships coexisted with coordination costs, unstable project participation, and partially unresolved responsibility boundaries. The contribution of governance innovation appeared to depend not simply on the number of actors involved, but on whether their relationships could be routinized, supported by adequate resources, and institutionally sustained.

#### 4.3.3 Embedding mutual aid in community governance

The evidence suggests that resident participation and neighborhood mutual aid could extend the reach of community services, particularly in addressing frequent but relatively low-intensity needs. Their contribution, however, was conditional rather than self-sustaining.

In F1, a volunteer representative indicated that some older residents benefited from having someone nearby to visit them, check on their situation, or notify community staff when assistance was needed (F1-VG-02). Such support contributed to companionship, everyday reminders, activity participation, basic information transmission, and the early identification of emerging needs. Nevertheless, participation was uneven across residents and remained partly dependent on organized projects, volunteer availability, and continued community mobilization. Volunteer involvement tended to be more active during project implementation but became more difficult to sustain after projects ended. In R1, an older resident explained that neighbors and village cadres were often the first people contacted when difficulties arose, because they lived nearby and were familiar with local households (R1-OR-03). A community staff member also noted that many younger family members worked outside the community, meaning that family care alone could not address all the everyday needs of older residents (R1-CS-02). These local relationships supported timely responses to routine needs such as home visits, meal assistance, reminders, companionship, information transmission, and initial help-seeking. At the same time, reliance on a relatively small number of village cadres, grid workers, active neighbors, and family members made service continuity vulnerable when these actors were unavailable or overburdened.

Mutual aid was therefore more applicable to high-frequency forms of support requiring relatively limited professional expertise than to continuous nursing, rehabilitation, chronic disease management, medical referral, or other specialized services. The evidence does not suggest that mutual aid was ineffective in R1. Rather, it indicates that mutual aid performed a meaningful but bounded function: it complemented formal services and strengthened local responsiveness but could not replace stable public provision and professional care capacity.

Taken together, the F1 and R1 cases suggest that local relational resources may enhance the reach and flexibility of care for older adults when they are linked to formal service providers, supported by continued community organization, and accompanied by clear referral arrangements for needs exceeding the capacity of neighborhood-based assistance.

#### 4.3.4 Building a governance closed loop

Case evidence suggests that a more integrated service process involved four interrelated stages. The first was demand identification, through which communities used household ledgers, routine visits, service records, and, where available, digital platforms to identify the differentiated needs of older residents. The second was supply organization, involving the connection of facilities, funding, service providers, volunteers, and local relational resources. The third was collaborative response, through which relevant actors were contacted or services were referred according to the identified need. The fourth was feedback and follow-up, through which service outcomes and changing needs could be recorded and used to adjust subsequent support.

When these stages were connected in routine practice, they appeared to reduce service fragmentation and support greater continuity. However, the completeness of this closed-loop process varied across cases. In particular, weak feedback arrangements, temporary project funding, insufficient professional providers, and unclear responsibility boundaries could interrupt the transition from demand identification to sustained service delivery.

In summary, governance innovation in the three cases involved the gradual reconfiguration of organizational relationships through multi-actor participation, community intermediation, social embedding, referral, and feedback. Such arrangements may help translate resource inputs into more accessible and continuous community support, but their practical effects remained dependent on institutional stability, organizational capacity, and sustained professional and financial support.

### 4.4 Smart technology in urban–rural care for older adults coordination

#### 4.4.1 Dynamic demand identification

Case evidence indicates that communities with stronger smart empowerment have generally established comprehensive digital registries for older adults. Through digital platforms, these communities are able to support classified management, service demand registration, task dispatch, and progress tracking.

In U1, field interview notes from community staff indicated that digital records were useful for identifying high-risk older adults, such as those living alone or requiring regular attention. However, the notes also suggested that these records needed to be updated frequently to remain useful in daily community work (U1-CS-04). This finding suggests that digital demand identification appeared more useful when data were regularly updated and connected to routine governance practices.

#### 4.4.2 Collaborative service response

The cases in which technology appeared more useful were not necessarily those with the most advanced infrastructure, but those in which platform information was more closely connected to routine governance and offline service delivery. Once a demand is identified, the system can dispatch tasks or referrals to the relevant service providers; medical institutions can conduct regular follow-up on the basis of health risk assessments; and community officials or grid managers can carry out home visits triggered by data alerts.

The field interview notes emphasized that platform information needed to be translated into offline action. In U1, interview notes from a medical worker indicated that health risk records on the platform could help medical personnel contact the community and arrange follow-up services more quickly (U1-MP-01). This finding shows that digital platforms can support collaborative responses when they are connected with medical and community service networks. In R1, field interview notes from a grid manager suggested that although the system could display some service needs, village cadres and grid workers still had to conduct visits, make phone calls, and help arrange services in practice (R1-CS-01). This finding indicates that smart technology does not replace human service provision; rather, it works through village cadres and grid-based coordination.

#### 4.4.3 From technology coverage to digital inclusion

Practical evidence further suggests that a prominent contradiction in Sichuan’s AFC development is that the groups most in need of support, including the oldest-old, persons with disabilities, and rural older adults, are often those with the weakest digital literacy. If communities prioritize online platforms and smart terminals while neglecting offline guidance, proxy assistance, and family support, efforts to improve efficiency may inadvertently generate new forms of exclusion. For technological platforms to function effectively, they must not only be understandable, accessible, and operable for older adults, but also be embedded within supportive social relationships. The contribution of digital platforms therefore appeared to depend on whether thresholds of use were reduced through offline guidance, proxy assistance, and support from community staff, volunteers, family members, and neighbors.

Field interview notes from older residents further illustrate why digital inclusion should be understood as a relational and organizational process. In U1, interview notes from an older resident indicated that although some older adults could use simple digital functions, they still needed help from community staff when the operation became more complicated (U1-OR-06). This finding shows that even in urban communities, smart services require accessible assistance. In R1, interview notes from another older resident suggested that small text on mobile phones, fear of making mistakes, and low confidence in using digital devices made some older adults prefer face-to-face assistance (R1-OR-02). This finding illustrates that digital exclusion is shaped not only by access to devices, but also by age-related usability barriers and confidence in technology use. Field interview notes from a volunteer in F1 further indicated that volunteers often helped older residents scan codes or make appointments, and that repeated assistance gradually reduced some older adults’ fear of using digital services (F1-VG-02). This finding suggests that digital inclusion is not only a technical issue, but also a process of accompaniment, learning, and trust-building.

## 5 Cross-case comparison and pathway differentiation

### 5.1 Urban embedded smart-service communities

The U1 case was characterized by a relatively strong digital foundation, comparatively developed organizational arrangements, and greater access to medical and social service resources. These conditions make them well suited to providing refined support for the oldest-old, older adults living alone, and those with chronic conditions through smart terminals, data platforms, and embedded service networks. The defining feature of this model is the integration of health monitoring, emergency response, meal and housekeeping assistance, cultural activities, and daily care into older adults’ everyday life scenarios through the combined support of smart platforms, grid-based governance, and community service hubs.

The strengths of this model lie in its rapid service response, high precision in matching supply with demand, and strong access to professional resources, enabling it to address the multilayered and dynamic care needs of older adults in high-density communities. However, its limitations are also evident. It depends on comparatively high levels of platform development, organizational management, and cross-actor coordination. As a result, its replication and wider diffusion require strong urban infrastructure and governance capacity as foundational conditions.

### 5.2 Urban–rural fringe mutual-aid communities

The F1 case combined a formal governance foundation with relatively strong neighborhood networks and traditions of mutual aid. The core characteristic of this model lies in its intermediary role in resource linkage and organizational transformation. Through community mobilization, resident deliberation, volunteer credit mechanisms, and the participation of social organizations, it integrates formal services with informal support and thereby appeared to support greater governance flexibility.

The strength of this model lies in its capacity to absorb and diffuse resources, forming a relatively smooth chain of service transmission between urban and rural areas. This pathway may be relevant to urban–rural fringe communities characterized by complex demographic structures, high population mobility, and the potential to cultivate community identity. Its limitation, however, is equally clear: without stable institutional interfaces and sufficient grassroots capacity to sustain these efforts, it is difficult to convert such initiatives into normalized and durable modes of operation.

### 5.3 Rural resource extension communities

Rural communities in the study faced substantial practical constraints, including dispersed populations, inadequate facilities, limited market participation, and low levels of digital literacy. The R1 case indicates that a feasible approach under these conditions involved establishing low-cost service nodes, reusing existing public spaces, strengthening village-level coordination, and extending county- or township-level resources downward. In practice, these arrangements included mobile health services, meal assistance, home visits, contracted services, neighborhood support, and basic communication functions supported by digital tools where available.

The potential contribution of this model lay in its ability to extend basic and frequently needed services within a resource-constrained setting. Its alignment with local social relationships also facilitated the identification of routine needs and initial responses. However, the model did not provide a complete substitute for professional care provision for older adults. Its operation remained dependent on external institutional support, village-level organizational capacity, and a limited number of active local actors. Without continued professional, financial, and organizational support, service continuity and quality could be difficult to maintain.

The R1 evidence therefore points to a bounded rather than universally transferable model. It may be relevant to rural communities with dispersed populations and limited resources, particularly for meal assistance, home visits, companionship, information transmission, and initial referrals. More specialized needs, including nursing, rehabilitation, chronic disease management, and complex care coordination, continued to require stable connections with professional institutions.

### 5.4 Common patterns and context-dependent pathways

The cross-case comparison suggests that the three communities shared several broad patterns, while the specific form and effectiveness of these patterns varied according to local context. Across U1, F1, and R1, existing resources appeared more likely to contribute to age-friendly outcomes when they were reorganized around accessible service sites, identifiable needs, and workable relationships among participating actors. Governance arrangements appeared more sustainable when coordination, referral, and feedback could be incorporated into routine community practices. Smart technology contributed most visibly when it supported, rather than replaced, interpersonal and offline service processes.

These similarities should not be interpreted as a universal causal mechanism. Instead, they point to a set of context-dependent relationships among resource availability, organizational capacity, technological embedding, and local social networks. U1 relied more heavily on digital platforms, grid-based governance, and professional service resources. F1 combined formal provision with neighborhood participation but remained vulnerable to project dependence and unstable institutional interfaces. R1 relied more strongly on village-level coordination, local relationships, and external resource extension, while facing limitations in professional service depth and continuity (Table 5).

**Table 5:** Comparison of context-dependent development pathways.

**Case**	**Resource allocation characteristics**	**Governance characteristics**	**Role of smart technology**	**Observed contribution**	**Observed limitations**
U1	Relatively resource-rich setting, developed platforms, and a comparatively high level of service embedding	Relatively developed grid-based governance and multi-actor coordination	May support demand identification, task dispatch, health follow-up, and service response	Relatively timely and differentiated service response within the urban case	High coordination workload; continuing digital exclusion among some older adults; dependence on data updating and offline assistance
F1	Coexistence of formal service resources, project resources, volunteer participation, and neighborhood mutual aid	Visible but uneven resident participation and project-sensitive collaboration	May facilitate information linkage, need identification, and service organization	Greater flexibility in responding to diverse and low-intensity needs	Participation may weaken after projects end; institutional interfaces and service continuity remain unstable
R1	Reuse of existing spaces and reliance on downward resource extension and localized support	Village-level coordination supported by local relationships and external institutions	Mainly provides basic communication, information transmission, and proxy-assisted access	May extend the reach of basic, frequent, and low-intensity services at relatively low cost	Limited professional service depth; dependence on a small number of local actors; vulnerability in continuous and specialized care

Rather than identifying a single optimal model, the comparison points to three context-dependent pathways. For urban communities similar to U1, greater attention may be directed toward integrating digital platforms with offline services and reducing coordination burdens. For urban–rural fringe communities similar to F1, the priority may lie in stabilizing the relationship between formal institutions, project-based providers, and informal mutual-aid networks. For rural communities similar to R1, county- and township-level resource extension may support a basic service network, but such arrangements also require professional referral channels and sustained external support. The typological comparison therefore suggests an analytical relationship rather than a fixed mechanistic law: resource conditions shape the range of feasible services, governance arrangements influence how resources are organized and sustained, and smart technologies may connect and amplify these processes when they are embedded in accessible offline support systems.

## 6 Conclusion and implications

Based on the evidence from the three anonymized cases, this study offers four main observations. First, the constraints encountered in the cases appeared to arise not only from resource shortages, but also from the interaction of spatial mismatch, fragmented service processes, digital exclusion, and incomplete governance coordination. The relative importance of these constraints varied across U1, F1, and R1. Second, resource allocation appeared more likely to improve service accessibility when existing spaces, professional capacity, community organizations, and external resources were reorganized around locally identified needs. The case evidence therefore suggests that increasing investment alone may be insufficient unless resources can be converted into usable and continuous community-level support. Third, governance innovation and smart technology appeared to contribute more effectively when they were connected. Multi-actor participation, referral, community intermediation, and feedback could support service continuity, while digital records and platforms could assist demand identification and response. However, these contributions remained conditional on data maintenance, offline assistance, clearly assigned responsibilities, stable funding, and the availability of professional providers. Fourth, the cross-case comparison points to a shared pattern rather than a universal development mechanism. Across the three cases, dispersed resources could potentially be translated into more accessible support through resource reorganization, governance coordination, and context-appropriate technological embedding. The form and effectiveness of this process nevertheless differed according to population structure, resource endowment, governance capacity, digital conditions, and local social relationships.

From the perspective of urban–rural integration, this study contributes to extending AFC research beyond a predominantly static focus on physical environment and service availability toward a more process-oriented consideration of resource mobility, governance coordination, and technological embedding. Rather than claiming statistical generalizability, the study provides an analytically transferable account of how these processes operated across three contrasting community contexts in western China. The comparison points to three context-dependent pathways: an urban embedded smart-service pathway, an urban–rural fringe mutual-aid coordination pathway, and a rural resource-extension pathway. These pathways should be understood as analytical types rather than prescriptive or universally applicable models. They offer policy considerations for communities with broadly comparable conditions, while local adaptation and further empirical assessment remain necessary.

Despite drawing on multiple data sources and adopting a comparative case design, this study has several limitations. First, the analysis was based on three communities in Sichuan Province. The findings should therefore be understood as analytically transferable rather than statistically generalizable, and their applicability to regions with different institutional, demographic, socioeconomic, and service-resource conditions requires further examination. Second, interview coverage was uneven across the three cases, particularly in R1, which may have limited the diversity of rural stakeholder perspectives represented in the analysis. Third, although 16 interviews were audio-recorded and transcribed verbatim, the remaining interviews were documented through detailed contemporaneous notes. Excerpts derived from these notes may not fully preserve the linguistic richness and nuances of participants’ original expressions. Finally, thematic development necessarily involved researcher interpretation. Potential subjectivity was mitigated through triangulation across data sources, coding review and researcher discussion, the maintenance of an audit trail, and systematic cross-case comparison.

Future research could include a broader and more diverse range of communities, expand the coverage of rural participants and stakeholder groups, and combine qualitative inquiry with quantitative assessment to examine the wider applicability of the identified mechanisms and proposed development pathways. Longitudinal designs would also be valuable for assessing whether project-based collaboration, mutual-aid participation, digital service arrangements, and resource-extension mechanisms can be sustained over time and how they affect service equity, continuity, user experience, and the well-being of older residents.

## Data Availability

The raw data supporting the conclusions of this article will be made available by the authors, without undue reservation.
